# Application of a high throughput Alamar blue biofilm susceptibility assay to *Staphylococcus aureus *biofilms

**DOI:** 10.1186/1476-0711-8-28

**Published:** 2009-10-27

**Authors:** Robin K Pettit, Christine A Weber, George R Pettit

**Affiliations:** 1 Cancer Research Institute and Department of Chemistry and Biochemistry, Arizona State University, Tempe, Arizona 85287, USA

## Abstract

**Background:**

*Staphylococcus aureus *and *S. epidermidis *biofilms differ in structure, growth and regulation, and thus the high-throughput method of evaluating biofilm susceptibility that has been published for *S. epidermidis *cannot be applied to *S. aureus *without first evaluating the assay's reproducibility and reliability with *S. aureus *biofilms.

**Methods:**

*Staphylococcus aureus *biofilms were treated with eleven approved antibiotics, lysostaphin, or Conflikt^®^, exposed to the oxidation reduction indicator Alamar blue, and reduction relative to untreated controls was determined visually and spectrophotometrically. The minimum biofilm inhibitory concentration (MBIC) was defined as ≤ 50% Alamar blue reduction and a purple/blue well 60 min after the addition of Alamar blue. Because all of the approved antibiotics had MBICs >128 μg/ml (most >2048 μg/ml), lysostaphin and Conflikt^®^, with relatively low MBICs, were used to correlate Alamar blue reduction with 2,3-bis(2-methoxy-4-nitro-5-sulfophenyl)-2*H*-tetrazolium-5-carboxanilide (XTT) reduction and viable counts (CFU/ml) for *S. aureus *ATCC 29213 and three clinical isolates. Alamar blue's stability and lack of toxicity allowed CFU/ml to be determined from the same wells as Alamar blue absorbances.

**Results:**

Overall, Alamar blue reduction had excellent correlation with XTT reduction and with CFU/ml. For ATCC 29213 and two clinical isolates treated with lysostaphin or Conflikt^®^, Alamar blue reduction had excellent correlation with XTT reduction (r = 0.93-0.99) and with CFU/ml (r = 0.92-0.98). For one of the clinical isolates, the results were moderately correlated for Conflikt^® ^(r = 0.76, Alamar blue vs. XTT; r = 0.81, Alamar blue vs. CFU/ml) and had excellent correlation for lysostaphin (r = 0.95, Alamar blue vs. XTT; r = 0.97, Alamar blue vs. CFU/ml).

**Conclusion:**

A reliable, reproducible method for evaluating biofilm susceptibility was successfully applied to *S. aureus *biofilms. The described method provides researchers with a simple, nontoxic, relatively inexpensive, high throughput measure of viability after drug treatment. A standardized biofilm Alamar blue assay should greatly increase the rate of discovery of *S. aureus *biofilm specific agents.

## Background

In the U.S., one million nosocomial infections each year are related to infections caused by biofilms on implanted devices [1]. Mortality for septicemias associated with vascular devices ranges from 20-40% [2], and intravenous catheters are the most common cause of nosocomial septicemia [3]. *Staphylococcus aureus *and *S. epidermidis *are the most common infectious agents associated with foreign device infections [4-6], and are found in biofilms in a wide range of other diseases, including endocarditis and osteomyelitis [7].

In vitro surface-associated and in vivo device-associated bacterial biofilms are generally quite resistant to antibiotic treatment [7,8]. Despite decades of research in this area, treatment options are limited. A factor contributing to this unmet need is the lack of a standardized method for determining the drug susceptibility of bacterial biofilms. Several methods are available, but are limited by long processing times, incompatibility with high throughput, expensive reagents or equipment, or the method measures mass instead of viability [9-13]. A simple high-throughput assay that measures viability has been standardized for *S. epidermidis *[14] and *Candida albicans *[15]. This colorimetric Alamar blue (AB) assay was reliable, reproducible and had good to excellent correlation with two other biofilm susceptibility methods, 2,3-bis(2-methoxy-4-nitro-5-sulfophenyl)-2*H*-tetrazolium-5-carboxanilide (XTT) reduction and viable counts (CFU/ml) for four strains of *S. epidermidis*.

Alamar blue has been used extensively in mammalian cell culture cytotoxicity assays and planktonic bacterial and fungal susceptibility assays, and is a simple, one-step procedure, in which metabolic activity results in the chemical reduction of AB. Alamar blue is reduced by FMNH_2_, FADH_2_, NAHD, NADPH and the cytochromes (product literature, Trek Diagnostic Systems). Alamar blue both fluoresces and changes color in response to chemical reduction, and the extent of the conversion is a reflection of cell viability (product literature, Trek Diagnostic Systems). Continued growth maintains a reduced environment while inhibition of growth maintains an oxidized environment. Alamar blue is water soluble, so the washing/fixation/extraction steps required in other commonly used cell proliferation assays are not required. Data may be collected with the naked eye, or for increased sensitivity, with either fluorescence-based or absorbance-based instruments. Alamar blue is also nontoxic to both the investigator and to the cells of interest, so it is safe to work with, easily disposed of and less likely to interfere with normal metabolism in test cells. In addition, AB is stable, so long incubations are possible, as are kinetic studies.

There are numerous differences in biofilm structure, growth and regulation in *S. aureus *and *S. epidermidis *[16-18]. Biofilms from different species of *Candida *also differ in structure and growth [19,20], so the colorimetric XTT susceptibility assay must be standardized for each species [21]. The high-throughput method of evaluating biofilm susceptibility that has been published for *S. epidermidis *cannot be applied to *S. aureus *without first evaluating the assay's reproducibility and reliability with *S. aureus *biofilms.

## Methods

### Strains

*Staphylococcus aureus *ATCC 29213 (wound isolate) and three clinical isolates (520009, 520016, 520020; Arizona Department of Health Services, Phoenix, AZ) were maintained on Mueller-Hinton agar (MHA) at 35°C. To confirm biofilm formation, *S. aureus *ATCC 29213, a known biofilm former [10], and the three clinical isolates were grown on glass and polystyrene for 24 and 48 h at 35°C without shaking, stained with Congo red, rinsed, drained and adhesion relative to 17 other clinical isolates was ranked.

### Antimicrobial agents

Clindamycin, bacitracin, vancomycin, ciprofloxacin, gentamicin, rifampin, nitrofurazone and enrofloxacin were obtained from ICN. Ceftriaxone, penicillin and oxacillin were from Sigma. Clindamycin, rifampin, nitrofurazone and enrofloxacin were dissolved in sterile dimethyl sulfoxide (DMSO). The remaining antibiotics and lysostaphin were dissolved in sterile H_2_O. Conflikt^® ^(Decon Laboratories Inc.) was used as received.

### Alamar blue biofilm susceptibility assay

The AB biofilm susceptibility assay and calculation of the percent reduction of AB were performed as described for *S. epidermidis *[14]. Alamar blue (Trek Diagnostic Systems), which is blue-colored in its oxidized state, is reduced in metabolically active cells to the pink colored resorufin. Alamar blue was aliquoted and stored at -80°C. Prior to each experiment, AB was brought to room temperature and vortexed. Exposure of AB to light was minimized throughout experiments. Isolated colonies from 18-22 MHA plates were used to prepare inocula. Assays were performed in flat bottom, polystyrene, non-tissue culture treated microtiter plates containing 5 × 10^5 ^CFU/ml in MHIIB media, with final well volumes of 100 μl. Plates were incubated at 37°C without shaking. Two fold-dilutions of drugs in cation-adjusted MHIIB were prepared external to the plates. After 24 h, 50 μl was removed from all experimental and control wells, and 50 μl of the appropriate drug dilution added. Biofilms were exposed to drugs for 20 h at 37°C without shaking. After 20 h, 5 μl AB was added to wells (105 μl total volume), the plates shaken gently and incubated for 1 h at 37°C. Plates were gently shaken again and absorbance at 570 nm and 600 nm obtained in a Perkin Elmer Wallac Victor^3 ^microplate reader. For experiments with multiple time points, plates were kept in a 37°C incubator between absorbance readings. Controls included media alone, media plus AB, media plus AB plus drug dilution, and cells plus media plus AB. Percent reduction of AB was calculated using the manufacturer's formula, with replacement of their negative control, which contains only media plus AB, with a more robust negative control, media plus AB plus a drug concentration equal to each experimental well:



where,

ε_ox _= molar extinction coefficient of Alamar blue oxidized form (blue)

ε_red _= molar extinction coefficient of Alamar blue reduced form (pink)

A = absorbance of test wells

A' = absorbance of negative control well

λ_1 _= 570 nm

λ_2 _= 600 nm

The AB minimum biofilm inhibitory concentration (MBIC) was defined as the lowest drug concentration resulting in ≤ 50% reduction of AB and a purple/blue well 60 min after the addition of AB [14]. Assays were performed at least twice, and the average % reduction used to determine the AB MBIC.

### Alamar blue planktonic susceptibility assay

Planktonic susceptibility testing of *S. aureus *was performed by the CLSI reference broth microdilution assay (BMA) [22] as previously described [14]. Assays were performed at least twice, and the average % reduction used to determine the MIC. The AB MIC was defined as the lowest drug concentration resulting in ≤ 50% reduction of AB and a purple/blue well 60 min after the addition of AB [14].

### Biofilm 2,3-bis(2-methoxy-4-nitro-5-sulfophenyl)-2H-tetrazolium-5-carboxanilide (XTT) reduction assay

The biofilm XTT reduction assay and calculation of percent formazan production were performed exactly as described for *S. epidermidis *[14].

### Biofilm CFU/ml assay

CFU/ml were obtained from the same wells used to obtain biofilm AB absorbances as previously described [14].

### Correlation of Alamar blue reduction to XTT reduction and CFU/ml

For correlation experiments, AB, XTT and CFU/ml assays were performed the same day using a single inoculum. Experiments were repeated at least twice. Pearson's two-tailed correlations were calculated with Prism 4 software using averaged data from the entire range of drug concentrations.

## Results

When the relative adhesion of 20 *S. aureus *clinical isolates and biofilm positive ATCC 29213 was scored, *S. aureus *ATCC 29213 and clinical isolates 520009, 520016 and 520020 ranked medium to high on both glass and polystyrene (three isolates were biofilm-negative on both substrates; the remaining isolates ranked medium to high on one or both substrates).

In order to make the assay as efficient as possible, the shortest possible AB reduction endpoint was determined as for *S. epidermidis *[14]. Twenty-four hour *S. aureus *biofilms were treated for 20 h with ciprofloxacin, lysostaphin or Conflikt^®^. Alamar blue was then added and absorbance determined 30, 60 and 90 min after the addition of AB. The susceptibility pattern was clear at 60 min (data not shown), just as with *S. epidermidis *[14], so this time point was chosen as the endpoint for absorbance readings in all experiments.

Prior to determining biofilm susceptibility, MICs for a variety of antibiotics with different mechanisms of action were determined for planktonic-grown *S. aureus *ATCC 29213 (Table 1). Turbidometric and AB MICs for planktonic grown strains were identical or within one twofold dilution (data not shown). MICs and MBICs were defined as the lowest drug concentration resulting in ≤ 50% reduction of AB, and in the case of noncolored compounds, a purple/blue well 60 min after the addition of AB (for colored compounds, AB reduction can only be determined spectrophotometrically). Alamar blue MBICs increased at least five-fold relative to planktonic AB MICs (Table 1), consistent with previous reports of many-fold increases in drug resistance of biofilm vs. planktonic-grown strains [7]. Even at doses of 2048 μg/ml, *S. aureus *ATCC 29213 biofilms were resistant to all antibiotics tested (Table 1). Nitrofurazone and ciprofloxacin could not be tested at doses higher than indicated (Table 1) because they precipitate at 512 μg/ml and 256 μg/ml, respectively.

**Table 1:** Alamar blue MICs and MBICs* of antibiotics against planktonic- and biofilm-grown *S. aureus *ATCC 29213

**Drug**	**Planktonic MIC (μg/ml)**	**Biofilm MBIC (μg/ml)**
gentamicin	0.25	>2048
clindamycin	16	>2048
bacitracin	32	>2048
nitrofurazone	8	>256
vancomycin	0.5	>2048
ciprofloxacin	0.25	>128
enrofloxacin	0.0625	>2048
ceftriaxone	2	>2048
penicillin	0.5	>2048
oxacillin	0.0625	>2048
rifampin	<0.0008	>2048

*MIC or MBIC defined as the lowest drug concentration resulting in ≤50% reduction of AB (average of two experiments) and a purple/blue well 60 min after addition of AB. MBICs for nitrofurazone and rifampin were only determined using the ≤50% reduction of AB criterion, as these antibiotics are colored at high doses.

**Figure 1:**
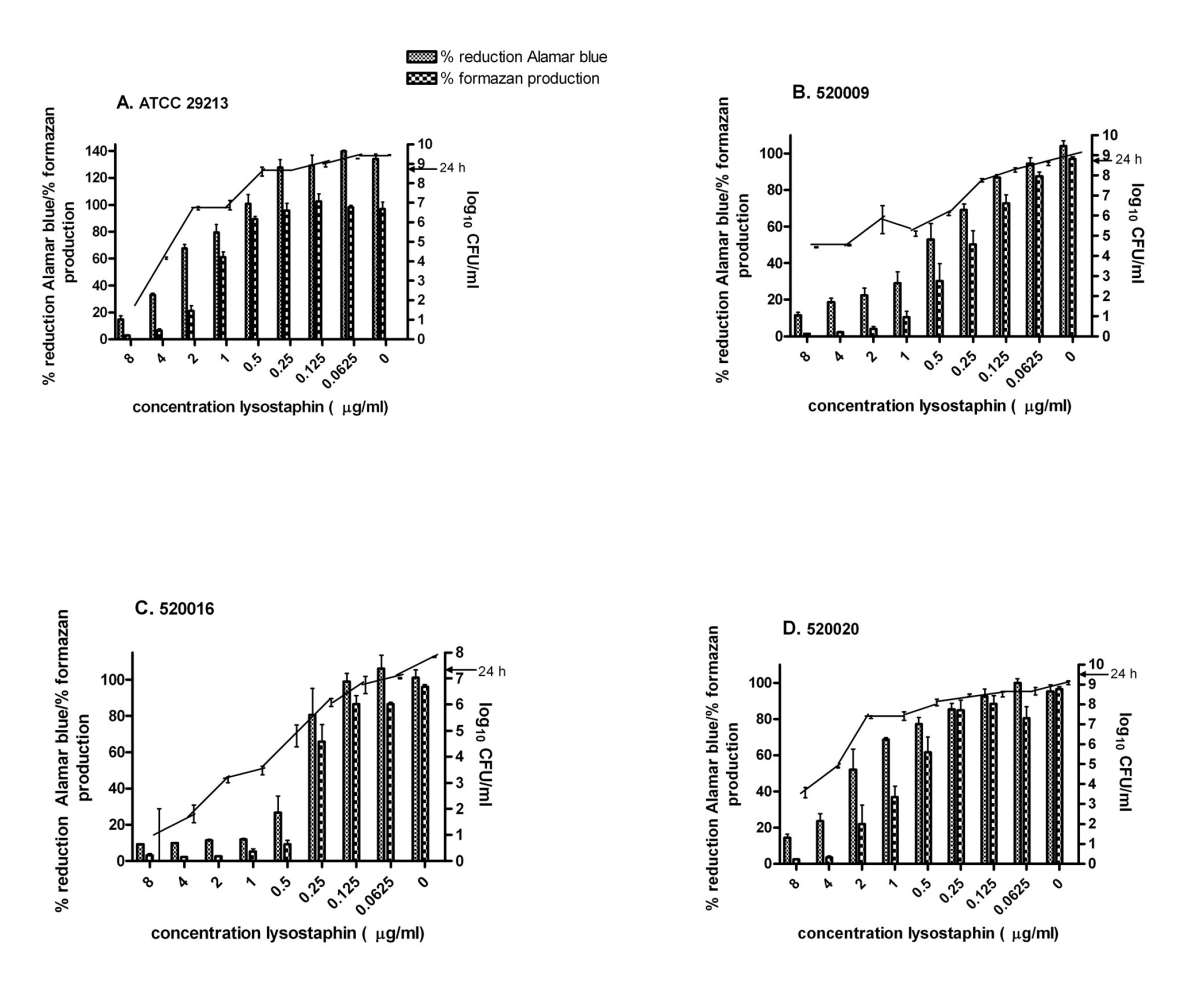
**Percent reduction of Alamar blue, percent formazan production and CFU/ml (right Y axis) for biofilms from four *S. aureus *strains (A-D) treated for 20 h with two-fold dilutions of lysostaphin**. Arrow, biofilm CFU/ml at 24 h (prior to drug treatment).

**Figure 2:**
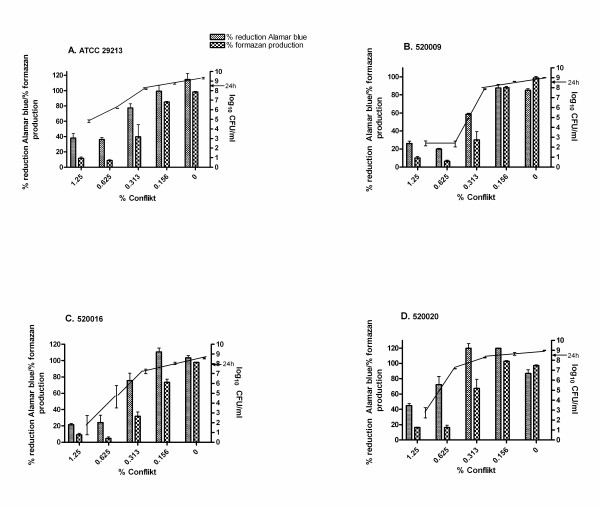
**Percent reduction of Alamar blue, percent formazan production and CFU/ml (right Y axis) for biofilms from four *S. aureus *strains (A-D) treated for 20 h with two-fold dilutions of Conflikt^®^**. Arrow, biofilm CFU/ml at 24 h (prior to drug treatment).

As no antibiotics inhibited *S. aureus *ATCC 29213 biofilms, we investigated an enzyme with reported activity against *S. aureus *biofilms, and a widely used disinfectant. Lysostaphin is an endopeptidase that cleaves the cross-linking pentaglycine bridges of the cell wall of staphylococci [23]. At low concentrations, lysostaphin kills *S. aureus *biofilms and, in addition, disrupts the biofilm matrix [24]. Coating catheters with lysostaphin prevents catheter colonization [25]. Conflikt^® ^(Decon) detergent disinfectant is a quaternary ammonium based disinfectant. Lysostaphin and Conflikt^® ^were effective against biofilms at much lower doses than the antibiotics (Figs. 1, 2). The AB MBIC for ATCC 29213 with lysostaphin, for example, was 4 μg/ml (Fig. 1A), and for Conflikt^® ^was 0.625% (Fig. 2A). Planktonic MICs for the four strains ranged from 2-4 μg/ml for lysostaphin, and from 0.039-0.078% for Conflikt^®^.

To help validate the described AB method for *S. aureus *biofilm susceptibility, AB assays were performed in parallel with XTT reduction assays, and CFU/ml were obtained from the same wells as AB absorbances. Reduction of tetrazolium salts, for e.g. XTT, is a common method of determining microbial cell viability [26], but XTT has several drawbacks, including toxicity. Alamar blue is stable and nontoxic, so unlike other metabolic assays, it is possible to plate directly out of wells over long periods for determination of viable cell counts. Similarly, in the CLSI planktonic assay [22], minimum bactericidal concentrations (MBCs) are obtained from the same wells as MICs. Biofilms of *S. aureus *ATCC 29213 and three clinical isolates were established overnight and treated with lysostaphin or Conflikt^® ^for 20 h. Alamar blue or XTT/menadione was added, and after 1 h, absorbances were obtained and wells scraped for dilution-plating (Figs. 1, 2). There was typically a small amount of biofilm growth from 24 h (Figs. 1, 2 arrows) to 44 h (Figs. 1, 2, 0 μg/ml). As evident in Figures 1 and 2, the AB MBIC corresponds to a many log-fold reduction in CFU/ml. These results support the hypothesis that metabolic activity is a useful measurement of viability, and that the AB assay is an extremely sensitive method.

Pearson's two-tailed correlation coefficients were calculated from the data in Figures 1 and 2. With the exception of Conflikt^®^-treated *S. aureus *520020, where AB vs. XTT and AB vs. CFU/ml were moderately correlated (r = 0.76, r = 0.81, respectively) [Table 2], AB had excellent correlation with XTT and CFU/ml (Table 2).

**Table 2:** Pearson's correlation coefficients for lysostaphin and Conflikt^®^-treated *S. aureus*

	**Pearson's correlation coefficients with:**			
	**Lysostaphin**		**Conflikt^®^**	
	
***S. aureus *strain**	**Almar blue vs. XTT**	**Alamar blue vs. CFU/ml**	**Alamar blue vs. XTT**	**Alamar blue vs. CFU/ml**
	
ATCC 29213	0.96	0.96	0.98	0.95
520009	0.99	0.98	0.96	0.97
520016	0.99	0.93	0.93	0.92
520020	0.95	0.97	0.76	0.81

## Discussion

Together, *S. aureus *and *S. epidermidis *are responsible for most implant and prosthetic device infections. As such, development of a standardized method to assess drug susceptibility of biofilms from both species is critical. An automated microtiter plate assay was recently described for susceptibility testing of *S. aureus *biofilms [27]. However, the assay was based on crystal violet staining, which does not measure viability. A high-throughput method employing AB, which does measure viability, has been described for *S. epidermidis *[14]. Recently, Peeters et al. [26] compared a variety of methods for quantifying biofilms (no susceptibility testing), and found the fluorescent AB assay to be very reliable for quantifying *S. aureus *biofilms. Alamar blue reduction data can be collected with the naked eye, or for increased sensitivity, with either fluorescence-based or absorbance-based instruments. Because absorbance-based instruments can be found in most laboratories, we evaluated the applicability of the AB method for susceptibility testing of *S. aureus *biofilms using absorbance. The biofilms produced by the four *S. aureus *isolates were so resistant to conventional antibiotics that lysostaphin and Conflikt^® ^had to be employed for correlation studies.

Although biofilm structure, growth and regulation in *S. aureus *and *S. epidermidis *is not equivalent [16-18], we have demonstrated that the AB assay is a reliable, reproducible method of evaluating biofilm drug susceptibility for both species [present study and [14]]. The standardization and correlation results presented here suggest that this method has promise for *S. aureus *biofilm susceptibility testing, but evaluation against a large panel of *S. aureus *strains is warranted. Certainly, it would be useful to have an effective conventional antibiotic for these larger studies, instead of widely reactive enzymes and detergents. Should AB be developed as a standard method of biofilm susceptibility testing, it would be useful to have consensus on the definition of the MBIC. While this and a previous study [14] used a value of ≤ 50% reduction of AB (which, as demonstrated, corresponds to a many log-fold reduction in CFU), much more data from a variety of labs will be necessary to define the most broadly applicable cutoff.

The benefits of AB over other methods of biofilm susceptibility testing are numerous and include simplicity, relative cost, compatibility with high throughput, lack of toxicity and importantly, AB measures viability, not simply mass. The major benefit of AB over XTT/menadione is its lack of toxicity (menadione has human toxicity), and the obvious benefit of AB over CFU/ml determination is its simplicity. The AB biofilm assay can be performed in any lab with a spectrophotometer, and is an excellent choice for high throughput labs. A standardized biofilm AB assay should greatly increase the rate of discovery of *Staphylococcus *biofilm specific agents.

## Competing interests

The authors declare that they have no competing interests.

## Authors' contributions

RKP designed the studies, performed the statistical analyses, and wrote the manuscript. CAW performed the studies and participated in data analyses. GRP arranged financial support and critically evaluated the manuscript. All authors read and approved the final manuscript.
